# Networks of Neuropsychological Functions in the Clinical Evaluation of Adult ADHD

**DOI:** 10.1177/10731911221118673

**Published:** 2022-08-29

**Authors:** Nana Guo, Anselm B. M. Fuermaier, Janneke Koerts, Oliver Tucha, Norbert Scherbaum, Bernhard W. Müller

**Affiliations:** 1 University of Groningen, The Netherlands; 2 University Medical Center Rostock, Germany; 3 Maynooth University, Ireland; 4 University of Duisburg-Essen, Germany; 5 University of Wuppertal, Germany

**Keywords:** adult ADHD, cognitive functions, network analysis, connections, centrality

## Abstract

This study applied network analysis to explore the relations between neuropsychological functions of individuals in the clinical evaluation of attention-deficit/hyperactivity disorder (ADHD) in adulthood. A total of 319 participants from an outpatient referral context, that is, 173 individuals with ADHD (ADHD group) and 146 individuals without ADHD (n-ADHD group), took part in this study and completed a comprehensive neuropsychological assessment. A denser network with stronger global connectivity was observed in the ADHD group compared to the n-ADHD group. The strongest connections were consistent in both networks, that is, the connections between selective attention and vigilance, and connections between processing speed, fluency, and flexibility. Further centrality estimation revealed attention-related variables to have the highest expected influence in both networks. The observed relationships between neuropsychological functions, and the high centrality of attention, may help identify neuropsychological profiles that are specific to ADHD and optimize neuropsychological assessment and treatment planning of individuals with cognitive impairment.

Attention-deficit/hyperactivity disorder (ADHD) is a neurodevelopmental disorder, characterized by symptoms of inattention, hyperactivity, and impulsivity (American Psychiatric Association, 2013). Up to 50% to 65% of individuals diagnosed with ADHD in childhood still present with ADHD symptoms in adulthood (Ebejer et al., 2012; Faraone et al., 2006; Fayyad et al., 2017). Numerous studies showed that adult ADHD, compared to typical development, is associated with a range of adverse outcomes, such as academic failure (Advokat et al., 2011; Arnold et al., 2020; Voigt et al., 2017), occupational underachievement (Fuermaier et al., 2021; Gjervan et al., 2012; R. Kaiser et al., 2008), problems in social relationships (Bruner et al., 2015; Michielsen et al., 2015; Paulson et al., 2005), sleep problems (Díaz-Román et al., 2018; Hvolby, 2015; Lugo et al., 2020), and lower quality of life (Agarwal et al., 2012; Quintero et al., 2019; Thorell et al., 2019).

Numerous neuropsychological studies demonstrated that adults with ADHD commonly present with impairments in multiple cognitive functions, including processing speed (Shanahan et al., 2006; Woods et al., 2002), selective attention (Butzbach et al., 2019; Onandia-Hinchado et al., 2021), sustained attention/vigilance (Marchetta et al., 2008; Salomone et al., 2020), memory (Alderson et al., 2013; Skodzik et al., 2017), planning (Desjardins et al., 2010; Fabio & Caprì, 2017), fluency (Onandia-Hinchado et al., 2021; O. Tucha et al., 2005), inhibition (Boonstra et al., 2010; Willcutt et al., 2005), and task switching (Cepeda et al., 2000; King et al., 2007). Impairments in these cognitive functions were still observed in adults with ADHD under stable psychopharmacological treatment (Fuermaier et al., 2017; Müller et al., 2007). However, not all adults with ADHD show impairments in all of these cognitive functions, which is often referred to as heterogeneity of cognitive performance in ADHD (Luo et al., 2019; Mostert et al., 2015; Seidman, 2006). Cognitive heterogeneity refers to the observation that, although individuals with ADHD typically present with impairments in attention and executive functions, not all patients with ADHD share the same type and degree of cognitive dysfunctions. Cognitive profiles of patients with ADHD range from individuals having no impairment in any of the cognitive functions assessed (Coghill et al., 2014; Guo et al., 2021; Mostert et al., 2015) to patients with ADHD disclosing marked cognitive impairments in all cognitive functions of a neuropsychological test battery (Luo et al., 2019; Seidman, 2006). Several pathway models of cognitive functions were proposed to address the issue of cognitive heterogeneity by suggesting that cognitive deficits of adults with ADHD mainly occur in two to six relatively independent neuropsychological functions (Coghill et al., 2014; Sonuga-Barke, 2003; Sonuga-Barke et al., 2010). For example, the dual pathway model suggests that executive deficits and delay aversion are two independent neuropsychological functions in which patients with ADHD frequently show impairments (Sonuga-Barke, 2003). The triple pathway model suggests that temporal processing may be a third neuropsychological domain (Sonuga-Barke et al., 2010) and the six-pathway model suggests there are six relatively independent neuropsychological functions, including inhibition, working memory, timing, delay aversion, decision-making, and response variability (Coghill et al., 2014). These conceptual studies on neuropsychological functions in adult ADHD provide support for the argument that cognitive deficits may exist relatively independently of each other. However, more recent studies provided empirical evidence that performances in the various cognitive functions in adult ADHD are not isolated but closely interrelated. For example, the performance of basic cognitive functions, such as processing speed and distractibility in tasks of basic attention, was shown to have a sizable effect on the performance of complex cognitive functions, such as inhibition, cognitive flexibility, and memory (Boonstra et al., 2010; Butzbach et al., 2019; Guo et al., 2021; Holst & Thorell, 2017; Mohamed et al., 2021). In addition, even though Coghill and colleagues (2014) claimed the existence of six relatively independent neuropsychological functions, the authors also reported significant and meaningful associations between these neuropsychological functions. Considering the inconsistency (e.g., independence or interrelatedness of cognitive functions) and limitations (e.g., limited cognitive functions assessed; small clinical samples, lack of replication, etc.) of previous studies, the relationship between the various cognitive functions still needs to be investigated to learn about existence and nature of a possible cognitive profile of adult ADHD.

An analytic technique for exploring the relationships between different variables, known as network analysis, may be a suitable approach to provide new insight into the picture of intertwined cognitive functions in adult ADHD. Compared to more traditional statistical approaches, such as univariate or multivariate group comparisons and correlation matrices, network analysis considers all variables for drawing a complex network that visually depicts the interrelations between variables. A network consists of nodes representing any conceivable variables (e.g., symptoms of mental disorders) and edges connecting these nodes which represent any conceivable relationship (e.g., correlation coefficients that indicate the degree of association between symptoms) (Borsboom & Cramer, 2013). Network analysis not only provides information about the correlations of various variables but also offers information about the relative importance of variables in the network by so-called node centrality indices, such as the node expected influence that has been most frequently used in recent studies. Nodes (or variables) with a high centrality may strongly affect other nodes in the network because of their strong connections (Bringmann et al., 2019; Opsahl et al., 2010). Network analysis has gained growing interest in the past decade for presenting complex relations in psychological science (Fonseca-Pedrero, 2017, 2018; Fried et al., 2017; McNally, 2016). A considerable number of studies applied network analysis in different psychological fields and clinical conditions, such as posttraumatic stress disorder (Cao et al., 2019; McNally et al., 2017; Peters et al., 2021), depression (Bringmann et al., 2015; Scott et al., 2021), anxiety (Beard et al., 2016; Fisher et al., 2017), and personality (Costantini et al., 2015; Richetin et al., 2017). In the field of ADHD, network analysis has been considered in a number of studies on the interaction between ADHD symptoms (Goh et al., 2020, 2021; Martel et al., 2016; Silk et al., 2019). For example, network analysis revealed that the various symptoms of ADHD contribute in different levels of importance to the clinical picture of ADHD, and this structure of symptoms may change in the development over time (Martel et al., 2016; Silk et al., 2019). Considering the advantages of network analysis in examining the relationships as well as the unique roles of a set of variables, we propose network analysis as a suitable approach to exploring the relationship between neuropsychological functions in ADHD, which may advance our understanding of cognitive profiles of adult ADHD.

This study is, to our knowledge, the first to apply network analysis on neuropsychological functions (performance test variables) of a large sample of clinically referred individuals at an ADHD outpatient clinic. The goal of this study is to examine the potential relationship between various aspects of cognitive functions of individuals diagnosed with or without ADHD. Specifically, this study aims to (a) explore the potential relationships between different cognitive functions of individuals diagnosed with ADHD as well as individuals who did not meet the diagnostic criteria of ADHD and whether there is a specific network structure for the ADHD group, which may have the potential to define ADHD-specific cognitive profiles. Moreover, this study aims to (b) define the centrality of cognitive functions, which is characterized by strong connections to numerous other cognitive functions. Central cognitive functions have the potential to be the primary targets of treatment to effectively improve the functioning of adults with ADHD.

## Methods

### Procedure and Participants

Participants were recruited from the ADHD outpatient clinic of the Department of Psychiatry and Psychotherapy, LVR-Hospital Essen, University of Duisburg-Essen, Essen, Germany. Individuals were referred for a diagnostic evaluation of ADHD because of being suspected of suffering from ADHD by their GPs, psychiatrists, or by themselves. All individuals underwent a comprehensive diagnostic evaluation of adult ADHD based on the *Diagnostic and Statistical Manual of Mental Disorders* (5th ed.; *DSM-5*; American Psychiatric Association, 2013). The diagnostic criteria for all individuals followed empirical-informed guidelines for the diagnosis of first-time ADHD in adulthood (Sibley, 2021), since information on a formal diagnosis of ADHD in childhood could not be retrieved reliably for all cases. The diagnostic evaluation consisted of a semistructured interview for the evaluation of ADHD and related psychopathology, self- and informant-report rating scales for symptoms and impairments, significant other reports, and consideration of objective indications of impairment in childhood and adulthood. In addition, all participants completed a comprehensive neuropsychological assessment. Even though neuropsychological assessments are part of the standard routine examination for all individuals referred to the ADHD outpatient clinic of the LVR-Hospital Essen, the results are not part of the standard diagnostic decision-making. Patients who were included in this study were assessed in 2020 and 2021. All individuals were informed about the scientific use of their data in anonymized form and gave written informed consent. Processing of their data for research purposes did not affect their clinical evaluation and treatment. This study received ethical approval from the ethical review board of the medical faculty of the University of Duisburg-Essen, Germany (20-9380-BO).

A total of 332 participants agreed to take part in this study; however, 13 of 332 participants were excluded from the data analysis. Eight participants were excluded because they were currently treated with psychostimulants at the time of the assessment. Another five participants were excluded as they were considered as not representative of this population, that is, individuals with mental disability (i.e., mental retardation, *n* = 2; fetal alcohol syndrome, *n* = 1), a neurological condition (i.e., dementia, *n* = 1), or a condition affecting the ability to perform cognitive tests (i.e., tic disorder, *n* = 1). Finally, a total of 319 participants were included in the data analysis, of which 173 participants received a diagnosis of ADHD after a comprehensive evaluation (ADHD group, *n* = 173) and 146 participants who did not meet the diagnostic criteria of ADHD (n-ADHD group, *n* = 146). In the *n*-ADHD group, 92 of the 146 participants did not reach diagnostic criteria of any psychiatric disorder, whereas 54 participants showed evidence for one or more other psychiatric disorders other than ADHD, including mood disorders (*n* = 34), personality disorders (*n* = 8), addiction disorders (*n* = 6), anxiety disorders (*n* = 4), adjustment disorders (*n* = 2), posttraumatic stress disorders (*n* = 2), eating disorders (*n* = 2), autistic disorders (*n* = 1), schizophrenia (*n* = 1), and somatization disorder (*n* = 1). In the ADHD group, 149 individuals were diagnosed with the predominantly combined symptom presentation, 23 individuals with the predominantly inattentive symptom presentation, and one individual with the predominantly hyperactive/impulsive presentation. Furthermore, 46 of the 173 patients with ADHD were additionally diagnosed with one or more comorbid psychiatric disorders (see Katzman et al., 2017, for a discussion of comorbidity in adult ADHD), including mood disorders (*n* = 27), addiction disorders (*n* = 11), personality disorders (*n* = 8), anxiety disorders (*n* = 6), adjustment disorders (*n* = 5), autistic disorders (*n* = 2), oppositional defiant disorders (*n* = 1), and posttraumatic stress disorders (*n* = 1). The observation that the distribution of psychiatric conditions other than ADHD was comparable and nonsignificantly different between the ADHD and the n-ADHD group (see Table 1), supports the notion that any potential group differences observed in test performances and network analysis are specific to ADHD. Demographic characteristics of all participants are presented in Table 1. No significant group differences were observed in age, *t* (283) = −1.780, *p* = .076, sex ratio, *χ*^2^ (1) = 3.781, *p* = .052, and education level, *χ*^2^(4) = 8.570, *p* = .073. As expected, patients with ADHD scored significantly higher in both current self-reported ADHD symptoms, *t* (299) = 3.398, *p* = .001, and retrospective self-reported ADHD symptoms for childhood, *t* (298) = 7.223, *p* < .001. However, no significant group difference was observed in self-reported cognitive functioning, *t* (313) = 1.922, *p* = .056.

**Table 1.** Demographic Characteristics of All Participants.

Demographic and clinical characteristics	ADHD (*n* = 173)	*n*-ADHD (*n* = 146)	*t*/*χ*^2^	*p* value	Cohen’s *r*^a^
Age (years)	33.2 ± 9.6	35.3 ± 11.2	−1.780	.076	0.10
Sex (male/female)	111/62	78/68	3.781	.052	0.11
Education level (% in 1/2/3/4/5)^b^	4.0/17.9/30.1/32.9/15.1	0/16.7/31.9/29.2/22.2^c^	8.570	.073	0.16
Current ADHD symptoms^d^	32.2 ± 9.5	28.3 ± 10.4	3.398	.001*	0.19
Childhood ADHD symptoms^e^	40.2 ± 13.3	29.5 ± 12.2	7.223	<.001*	0.38
Self-report cognitive functions^f^	77.8 ± 20.2	73.4 ± 20.4	1.922	.056	0.11
Psychiatric disorders other than ADHD (% of individuals with/without)					
Mood disorders	15.6/84.4	23.3/76.7	3.02	.082	0.10
Addiction disorders	6.4/93.6	4.1/95.9	0.795	.373	0.05
Personality disorders	4.6/95.4	5.5/94.5	0.122	.727	0.02
Anxiety disorders	3.5/96.5	2.7/97.3	0.138	.710	0.02
Adjustment disorders	2.9/97.1	1.4/98.6	0.853	.356	0.05
Autistic disorders	1.2/98.8	0.7/99.3	0.189	.664	0.02
Oppositional defiant disorders	0.6/99.4	0/100	0.847	.358	0.05
Post-traumatic stress disorder	0.6/99.4	1.4/98.6	0.533	.465	0.04
Eating disorders	0/100	1.4/98.6	2.385	.123	0.08
Schizophrenia	0/100	0.7/99.3	1.189	.276	0.06
Somatization disorder	0/100	0.7/99.3	1.189	.276	0.06

*Note.* ADHD = attention-deficit/hyperactivity disorder.

a Based on *Cohen’s* criteria for *r*: 0.1 indicates a small effect, 0.3 indicates a medium effect, and 0.5 indicates a large effect. ^b^ Education level (1/2/3/4/5) = no school-leaving qualification/compulsory school or secondary school completed/completed technical school or vocational training/higher school with university entrance qualification/university or college degree. ^c^ Education level was not reported in two cases. ^d^ Current ADHD symptoms were assessed by the German version of the ADHD self-report scale. ^e^ Childhood ADHD symptoms were assessed by the German version of the Wender-Utah Rating Scale–Short Version. ^f^ Self-report cognitive functions were assessed by the Questionnaire on Mental Ability of the Vienna Test System.

* Statistically significant at *p* < .01.

### Measures

#### Self-Report Scales for ADHD Symptoms and Cognitive Functions

The German short version of the Wender-Utah Rating Scale (WURS-K) was used to retrospectively assess ADHD symptoms in childhood (Retz-Junginger et al., 2003). A total of 25 items scored on a 5-point Likert-type scale were included in the WURS-K. The German version of the ADHD symptoms self-report scale (ADHD-SR) was administered to check the current ADHD symptoms (Rösler et al., 2004). A total of 18 items scored on a 4-point Likert-type scale were included in the ADHD-SR. The Questionnaire on Mental Ability (FLEI) of the Vienna Test System (VTS; Schuhfried, 2013) was used to measure self-reported cognitive functions. A total of 35 items assessed on a 5-point Likert-type scale were included. A sum score was calculated for each scale.

#### Neuropsychological Assessment

A computerized neuropsychological test battery for the assessment of cognitive functions in adult ADHD (CFADHD; L. Tucha et al., 2013) of the VTS was administered to all participants. The test battery was designed for clinical use to be sensitive to reveal cognitive deficits in adult ADHD and was not composed for research purposes tailored to this study. Because of this naturalistic setting, not all cognitive functions discussed in the literature review were assessed on the present patient samples.

##### Selective Attention

The Perceptual and Attention Functions—selective attention (WAFS; Sturm, 2011) was used to measure selective attention. In this test, a total of 144 geometric stimuli (triangle, circle, and square) that may get darker or lighter or stay the same were presented to the participants. Participants were asked to react to 30 target stimuli (i.e., a circle becomes darker, a circle becomes lighter, a square becomes darker, and a square becomes lighter) by pressing the response button as quickly as possible and ignoring distracting stimuli. Recorded outcome measures included reaction time (RT) in milliseconds and dispersion of reaction time (SDRT), as well as the number of omission errors. The internal consistency (Cronbach’s α) of the main variables was reported to be 0.95.

##### Vigilance

Vigilance was assessed with the Perceptual and Attention Functions—vigilance (WAFV; Sturm, 2012). In this test, a total of 900 squares that sometimes get darker were presented to the participants. The participants had to react to 50 target stimuli (square becomes darker) by pressing the response button as fast as possible and ignoring other distracting stimuli. The mean RT in milliseconds and the number of omission errors were registered. The internal consistency (Cronbach’s α) of the main variables was reported to be 0.96.

##### Working Memory

Working memory was measured with the 2-back design of the NBV (N-Back Verbal; Schellig & Schuri, 2012) task, which was developed by Kirchner (1958). In this task, a succession of 100 consonants was presented one by one to the participants who had to press the response button if the consonant currently displayed was identical to the last-but-one consonant and ignored it if it was not. The number of correct responses was recorded. The internal consistency (Cronbach’s α) of the main variable correct responses was reported to be 0.85.

##### Figural Fluency

The 5-point test—Langensteinbach Version was administered to measure figure fluency (Rodewald et al., 2014). In this test, participants were presented with five symmetrically arranged dots (like the number five on a dice) and were asked to create as many unique patterns as they can in 2 minutes by connecting at least two dots. The number of unique patterns created in 2 minutes was recorded. The internal consistency (Cronbach’s α) of this variable was reported to be 0.86.

##### Interference Control

Interference control was assessed with the Stroop Interference Test (Schuhfried, 2016), which was developed by Stroop (1935). This test form included two baseline conditions and two interference conditions. The first baseline condition was the reading-baseline condition, in which color-words (RED, GREEN, YELLOW, BLUE) printed in gray were presented to participants who were asked to press the button with the same color as the meaning of the presented color-word. The second baseline condition was the naming-baseline condition, in which banners printed with four colors (red, green, yellow, and blue) were presented to participants who had to press the button with the same color as the color of banners. The first interference condition was the reading-interference condition, in which color-words printed in mismatching ink (e.g., RED printed with green ink) were presented to participants who were asked to press the button with the same color as the meaning of the color-word while ignoring the ink of it. The second interference condition was the naming-interference condition, which was different from the reading-interference condition, where participants were asked to press the button with the same color as the ink of the color-word while ignoring the meaning of it. Participants were asked to react as fast as possible throughout the test. The variables of interest were reading interference and naming interference. *Reading interference* was calculated by subtracting the time needed for the reading-baseline condition from the time needed for the reading-interference condition. *Naming interference* was calculated by subtracting the time needed for the naming-baseline condition from the time needed for the naming-interference condition. The internal consistency (Cronbach’s α) of the main variables was reported to be 0.97.

##### Processing Speed and Cognitive Flexibility

The Trail-Making Test—Langensteinbach Version (TMT-L; Rodewald et al., 2012) was used as a measure of processing speed and cognitive flexibility. In part A, 25 numbers (1–25) were simultaneously presented on the computer screen and participants had to connect the numbers as fast as possible in ascending order. In part B, there were 13 numbers (1–13) and 12 letters (A–L), and participants were asked to connect numbers and letters alternately and in ascending order as quickly as possible. The time needed for part A (in seconds) was used as a measure of processing speed and the time needed for part B was used as a measure of cognitive flexibility. The internal consistency (Cronbach’s α) of part A and part B was reported to be 0.92 and 0.81, respectively.

##### Planning

The Tower of London—Freiburg Version (TOL-F; Christoph et al., 2011) was administered to assess planning ability. In this task, there were three rods of different heights, on which three differently colored balls (yellow, red, and blue) were placed. The left-hand rod can hold three balls, the central rod can hold two balls, and the right-hand rod can hold one ball. Start state and goal state, as well as the minimum number of moves needed to convert the start state into the goal state, were presented on the screen. Participants had to convert the start state into the goal state by the minimum number of moves in 60 seconds. The next item was presented automatically as soon as the current item had been solved in 60 seconds or the current item was not solved after 60 seconds. This test consisted of 28 items, comprising four three-move items and each eight four-move, five-move, and six-move items. These items were presented to participants in the order of an increasing minimum number of moves. The items at the start of the test that can be solved in three moves served as practice items. The number of the four- to six-move items solved in the minimum number of moves was recorded as the measure of planning ability. The internal consistency (Cronbach’s α) of this test was reported to be 0.70.

##### Response Inhibition

The Go/No-Go test paradigm (S. Kaiser et al., 2016) was used to measure response inhibition. In this test, a series of triangles (202) and circles (48) were presented one by one on the computer screen. Participants had to press the response button when triangles (Go trials, 80.8% of all trials) were presented and no response was required to circles (No-Go trials, 19.2% of all trials). The number of commission errors was registered. The internal consistency (Cronbach’s α) of this test was reported to be 0.83.

##### Task Switching

Task switching was measured with the SWITCH (Gmehlin et al., 2017). In this test, a series of bivalent stimuli which can be categorized based on form (triangle/circle) and brightness (gray/black) were presented. Participants were asked to react interchangeably based on these two dimensions (triangle/circle or gray/black). After each two items, the dimensions to which participants had to react changed. The items that require a reaction based on the same dimension as the preceding item were defined as repeated items, whereas the items that require a reaction based on the different dimension than the preceding item were defined as switch items. The variable of interest was *task switching accuracy*, which was the difference between the percentage of correct responses for switching and repeated tasks. The internal consistency (greatest lower bound) of this variable was satisfactory and reported to be 0.81.

### Statistical Analysis

Descriptive statistics and inferential group comparisons were computed using IBM SPSS (Version 25.0 for Windows). Network analyses of neuropsychological functions were performed with RStudio (RStudio Team, 2021).

#### Descriptive Statistics and Group Comparisons

The Median and interquartile range (IQR) of each test variable, as well as the percentage of individuals showing impairment in each of the neuropsychological functions, are presented in descriptive statistics. The interpretation of test data was based on norm scores provided by the test publisher. Impairment was defined if a test variable indicated a score equal to or below the 16th percentile (i.e., one standard deviation below the mean) of the respective normative group (Schuhfried, 2013). Furthermore, because our data are not normally distributed, test performances of groups were compared using nonparametric statistics (i.e., Mann–Whitney *U* tests). To control for alpha error growth in multiple testing, a stringent significance level of *p* < .01 was applied. Finally, the magnitude of group differences was indicated by the effect size Cohen’s *r*, with *r* = 0.1 indicating a small effect, *r* = 0.3 indicating a medium effect, and *r* = 0.5 indicating a large effect (Cohen, 1988).

#### Network Estimation

The networks of neuropsychological functions were estimated for the ADHD group and the n-ADHD group, using the R packages *bootnet* and *qgraph* (Epskamp et al., 2012, 2018). In these networks, 14 variables were depicted as nodes, and the partial correlation coefficients between neuropsychological functions were depicted as edges. Partial correlation coefficients represent the correlation between two variables after controlling for all other variables in the network (Borsboom & Cramer, 2013). To avoid spurious connections and make networks more interpretable, the graphical lasso algorithm, which is a variant of the prominent regularization algorithm least absolute shrinkage and selection operator (LASSO) was applied to estimate the network (Epskamp & Fried, 2018; Tibshirani, 1996). This graphical lasso algorithm controls the degree of regularization by a tuning parameter (*λ*), which can be determined using the Extended Bayesian Information Criterion (EBIC) (Chen & Chen, 2008; Friedman et al., 2008). The visualization of these networks was based on the Fruchterman–Reingold algorithm (Fruchterman & Reingold, 1991). In the graph that is plotted based on the Fruchterman–Reingold algorithm, nodes with stronger connections are placed more proximal to each other, and connections between nodes with higher absolute coefficients are represented with thicker and more saturated colored edges. In addition, identical layouts of nodes were produced for two groups using the *averageLayout* function of *qgraph* package to enable visual comparison between groups (Epskamp et al., 2012). As our data were not normally distributed, a rank transformation (Spearman correlations as input) (Isvoranu & Epskamp, 2021) was performed before estimating network structures.

#### Node Centrality Estimation

The relative importance of variables in the network was examined with node expected influence, which is a node centrality index representing the sum of connections for one node. Compared to node strength, which is the previously most used node centrality index representing the sum of the absolute value of connections for one node, node expected influence considers both positive and negative connections (Opsahl et al., 2010; Robinaugh et al., 2016). The *centrality, centralityTable*, and *centralityPlot* function of *qgraph* package was used to compute and plot the expected influence (Epskamp et al., 2012).

#### Accuracy and Stability Estimation

The accuracy of edge weights and the stability of the order of node centrality were examined. The edge weight accuracy was estimated by bootstrapping the 95% confidence intervals (CIs) of the edge weights, with smaller CIs indicating higher accuracy of the order of most edges in the network. Node centrality stability was estimated using the correlation stability coefficient (CS coefficient). Based on the simulation design of Epskamp et al. (2018), CS coefficients > 0.25 indicate moderate stability and >0.5 indicate strong stability. The R package *bootnet* was used to perform these analyses.

#### Network Comparison

The global connectivity strength of the network, which represents the sum of the weights of all edges within the network, was compared between the ADHD group and the n-ADHD group. The Network Comparison Test (NCT), which is a statistical testing procedure for network comparison, was used to perform these comparisons (van Borkulo et al., 2022). NCT compares the global connectivity strength of different group networks using the permutation test that repeatedly estimates the networks for randomly regrouped individuals and then calculates the accompanying test statistic. The R package *NetworkComparisonTest* was used for these comparisons (van Borkulo, 2018).

#### Additional Network Analyses

First, additional network analysis was performed on the individuals with the combined symptom presentation only to examine the potential influence of different symptom presentations of ADHD. Second, to address the possibility that different weights for each test (e.g., more variables were extracted from tests for selective attention and vigilance compared to other tests) may bias our findings, additional network analysis was performed based on averaged *Z*-scores per neuropsychological function. Finally, considering the nearly significant sex difference between the ADHD and n-ADHD group (*p* = .052), additional network analyses were carried out to examine the potential influence of sex.

## Results

### Descriptive Statistics and Group Comparisons

Descriptive statistics and group comparisons of neuropsychological test performance for the ADHD and n-ADHD groups are presented in Table 2. Compared to test norms, the number of individuals with an impairment in each test variable ranged from 9.9% to 57.9% in the ADHD group and from 10.4% to 42.4% in the n-ADHD group. Per test variable, the largest impairment rates were found in vigilance—omission errors (57.9% and 42.4% in the ADHD and n-ADHD group, respectively), response inhibition—commission errors (45.9% and 40.3%), and selective attention—SDRT (40.1% and 39.6%). The fact that impairment rates were at or below 16% of several test variables indicates that individuals with ADHD did not show decreased performance compared to normative data in a range of aspects of cognitive functioning. Data analyses indicate that the differences in impairment rates between two groups are small, and only the difference in the number of omissions of the vigilance test turned statistically significant (*p* = .003), as individuals in the ADHD group made significantly more omissions errors than the n-ADHD group as shown in a small effect of Cohen’s *r* = 0.16. Per neuropsychological function, the largest impairment rates were found in vigilance (58.5% and 44.4% in the ADHD and *n*-ADHD group, respectively), selective attention (55.2% and 55.5%), response inhibition (45.9% and 40.3%), and interference control (40.7% and 39.6%). Impairment in a given function is defined if impairment was observed in at least one test variable of this function.

**Table 2.** Neuropsychological Test Performance of the ADHD and n-ADHD Group.

Neuropsychological variables	ADHD (*n* = 173)			*n*-ADHD (*n* = 146)			Group comparison		
	Median	IQR	% impaired^a^	Median	IQR	% impaired^a^	*Z*	*p*	Cohen’s *r*^b^
Selective attention^c^—RT	347.00	87.25	16.37	355.00	95.75	13.99	−0.419	.675	0.02
Selective attention^c^—SDRT	1.26	0.11	40.12	1.22	0.12	39.58	−0.065	.948	0.003
Selective attention^c^—Omissions	0	1.00	32.56	0	1.00	28.67	−1.049	.294	0.05
Vigilance^d^—RT	446.00	123.00	20.47	443.00	109.50	14.69	−0.212	.832	0.01
Vigilance^d^—Omissions	2.00	5.00	57.89	1.00	3.00	42.36	−2.953	.003*	0.16
Working memory^e^	12.00	4.00	17.44	12.00	4.00	14.58	−0.766	.444	0.04
Figural fluency^f^	28.00	16.00	13.95	24.00	14.50	15.97	−2.226	.026	0.13
Interference control^g^—reading	0.17	0.15	31.40	0.17	0.15	29.86	−0.883	.377	0.05
Interference control^g^—naming	0.11	0.14	17.44	0.11	0.11	17.36	−0.773	.439	0.04
Cognitive flexibility^h^	28.15	12.68	12.28	29.00	11.90	11.80	−0.666	.505	0.04
Planning^i^	14.00	4.25	10.59	14.00	5.00	11.27	−0.915	.360	0.05
Response inhibition^j^	14.00	11.25	45.93	13.00	11.00	40.28	−1.572	.116	0.09
Task switching^k^	3.00	5.25	20.35	3.00	6.00	18.06	−0.032	.975	0.002
Processing speed^l^	18.40	6.60	9.88	18.80	5.55	10.42	−1.474	.141	0.08

*Note.* ADHD = attention-deficit/hyperactivity disorder; IQR = interquartile range; RT = reaction time; SDRT = dispersion of reaction time.

a Impairment defined if percentile rank ≤ 16; ^b^ Based on *Cohen’s* criteria for *r*: 0.1 indicates a small effect, 0.3 indicates a medium effect, and 0.5 indicates a large effect; ^c^ Perceptual and Attention Functions—selective attention (WAFS); ^d^ Perceptual and Attention Functions—vigilance (WAFV); ^e^ 2-back design of the N-Back Verbal (NBV); ^f^ 5-point test; ^g^ Stroop Interference Test; ^h^ Trail-Making Test, part B (TMT-B); ^i^ Tower of London—Freiburg Version (TOL-F); ^j^ Go/No-Go; ^k^ SWITCH; ^l^ Trail-Making Test, part A (TMT-A).

* Statistically significant at *p* < .01.

### Network Estimation

**Figure 1.**
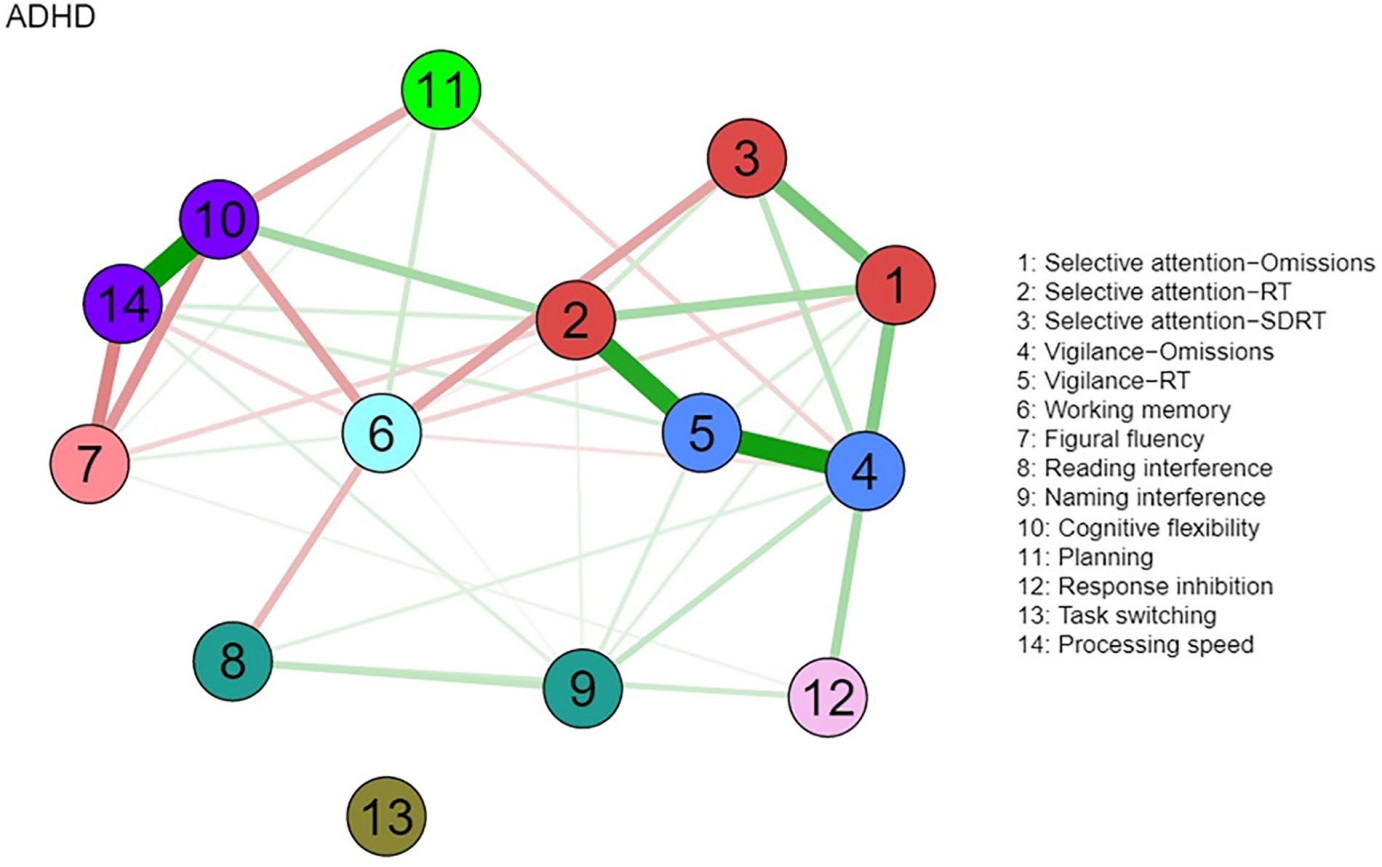
Network of Neuropsychological Functions for the ADHD Group (N = 173). *Note.* Nodes represent neuropsychological test variables. Neuropsychological test variables stemming from the same test are presented in the same color. Edges connecting nodes represent the regularized partial Spearman correlations. Higher absolute correlations are represented with thicker and more saturated colored edges. Green edges indicate positive correlations, and red edges indicate negative correlations. ADHD = attention-deficit/hyperactivity disorder; RT = reaction time; SDRT = dispersion of reaction time.

**Figure 2.**
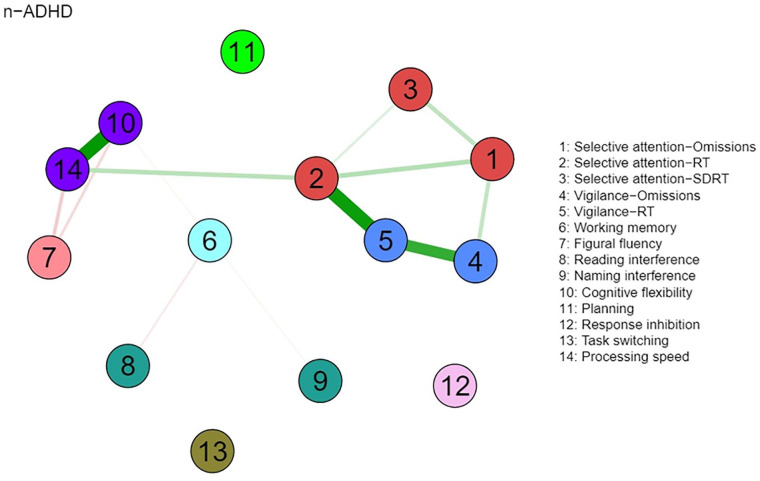
Network of Neuropsychological Functions for the n-ADHD Group (N = 146). *Note.* Nodes represent neuropsychological test variables. Neuropsychological test variables stemming from the same test are presented in the same color. Edges connecting nodes represent the regularized partial Spearman correlations. Higher absolute correlations are represented with thicker and more saturated colored edges. Green edges indicate positive correlation, and red edges indicate negative correlations. ADHD = attention-deficit/hyperactivity disorder; RT = reaction time; SDRT = dispersion of reaction time.

The visualized networks of the ADHD and n-ADHD groups are presented in Figures 1 and 2, respectively. For the ADHD group, a density network is depicted that connects almost all variables in the network. The *n*-ADHD network has few connections and most of the connections are weak. But the strongest connections appear to be consistent between these two networks, including the connections between two attention tests, that is, selective attention test and vigilance test (2, 4, and 5), as well as the connection between two variables stemming from the Trail-Making Test (10 and 14). Furthermore, connections between all variables of attention (selective attention and vigilance, see 1–5) and connections between figural fluency, cognitive flexibility, and processing speed (7, 10, and 14) were also observed in both the ADHD and *n*-ADHD networks. In addition, the connection between selective attention and working memory (3 and 6) as well as the connection between vigilance and response inhibition (4 and 12) was observed in the ADHD network. Compared to other variables, task switching (13) is relatively isolated in the ADHD network.

### Node Centrality Estimation

**Figure 3.**
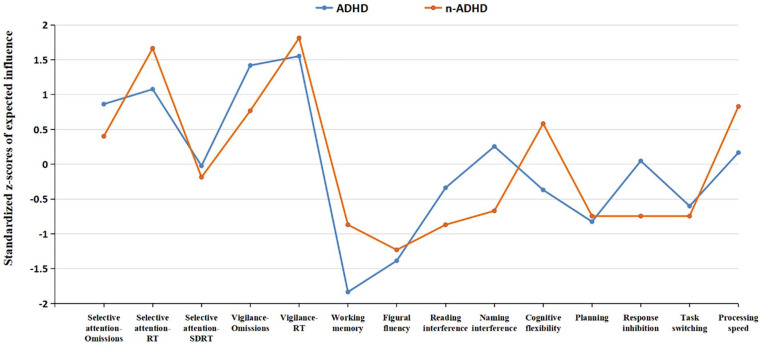
Node Expected Influence for the ADHD and n-ADHD Group. *Note.* Higher standardized *z*-scores indicate higher expected influence, and nodes with higher expected impact have closer and stronger relationships with other neuropsychological test variables in the network. ADHD = attention-deficit/hyperactivity disorder; RT = reaction time; SDRT = dispersion of reaction time.

Node centrality estimations are presented in Figure 3. In both groups, nodes with high expected influence are mainly attention-related variables, especially the RT of these tests. In the ADHD group, the nodes depicting the RT of selective attention, RT of vigilance, and omissions of vigilance task have the highest expected influence. In the n-ADHD group, the RT of selective attention and RT of vigilance have the highest expected influence.

### Stability Estimation

The edge weight accuracy estimation revealed moderate CIs and indicated that the orders of edge weights were accurately estimated in both the ADHD network and the n-ADHD network (see Figures S1 and S2 in Supplemental Material). The node centrality estimation revealed that the CS coefficients were 0.36 (ADHD) and 0.44 (n-ADHD), indicating that the orders of node centrality were stable.

### Network Comparison

The visual comparison suggests denser and stronger connections in the ADHD network than in the n-ADHD network. Further NCT analysis indicates that the global connectivity strength of the ADHD network is significantly stronger than that of the n-ADHD network (4.09 vs. 1.54, *s* = 2.55, *p* = .026).

### Additional Network Analyses

First, no meaningful differences were observed between network analysis based on the combined symptom presentation only and the analysis of the entire group of patients with ADHD. Second, the results of network analysis based on averaged *Z*-scores per neuropsychological function were comparable to our initial analysis. Finally, no significant differences were observed by sex. Please find detailed results in Supplemental Material.

## Discussion

This study analyzed neuropsychological performance data of a large sample of individuals at clinical evaluation of adult ADHD using both traditional descriptive and inferential statistics as well as network analyses to explore the relationship between neuropsychological functions. Traditional statistics showed neuropsychological impairments in a large proportion of both individuals in the ADHD and n-ADHD groups as compared to test norms, including deficits in vigilance, selective attention, inhibition, and reading-interference control. The marked impairments of neuropsychological functions observed in both groups support the earlier argument that neuropsychological assessment plays an important role in acquiring a comprehensive understanding of an individual’s cognitive strengths and weaknesses (Lange et al., 2014; Mapou, 2019; Seidman, 2006). Furthermore, the present data show that neuropsychological functions did not differ significantly between the groups in most of the measures, except for decreased vigilance performance (i.e., more omission errors) in the ADHD group. Pronounced vigilance impairments in the ADHD group underline the prominent role of vigilance and sustained attention tests in the clinical evaluation of ADHD, as it was demonstrated in a large body of empirical research on individuals with ADHD of various age groups (Bijlenga et al., 2019; Hall et al., 2016; Marchetta et al., 2008; Slobodin et al., 2020; L. Tucha et al., 2017) and advocated in an international consensus report (Fuermaier et al., 2018). Furthermore, the present neuropsychological performance data are also in line with prior research on an independent data set from the same referral context (Guo et al., 2021), stressing that cognitive deficits are not specific to individuals diagnosed with ADHD but occur commonly in individuals of this referral context. These results also corroborate earlier studies indicating that the assessment using neuropsychological tests may have limited value in the discrimination of individuals with ADHD from individuals with other psychiatric conditions (Barkley, 2019; Holst & Thorell, 2017; Pettersson et al., 2018).

A visual inspection of the networks displays an interrelated pattern of neuropsychological test performance in the ADHD group, with the strengths of the connections varying from weak to strong. Few connections were observed in the n-ADHD group, with significantly weaker global connectivity strength compared to the ADHD group. This finding gives an indication that the extensive connections between various neuropsychological functions are not uniform in all individuals in the clinical evaluation of adult ADHD. Most of the neuropsychological functions were connected in the ADHD group but were relatively isolated in the n-ADHD group. The finding that a denser and stronger network of neuropsychological functions was observed in the ADHD group compared to the n-ADHD group may provide further information for the cognitive profiles of adults with ADHD. For example, dense connections observed in the ADHD network indicate close relationships between cognitive functions, forming a holistic cognitive function system in individuals with ADHD (Karyakina & Shmukler, 2021). Furthermore, it could be speculated that the connected cognitive functions observed in the ADHD group may reflect a functional compensatory mechanism as it was suggested in functional imaging studies (Abramov et al., 2019; Hale et al., 2014). Compared to individuals who do not meet the diagnostic criteria of ADHD, individuals diagnosed with ADHD may compensate with the involvement of additional cortical areas to increase specific cognitive task performance (Fassbender & Schweitzer, 2006). This compensatory mechanism may lead to an inter-related involvement of cognitive functions and may result in close connections of cognitive functions in network analysis. Future studies using functional magnetic resonance imaging would be suited to further explore the relationship between neuropsychological functions and compensatory mechanisms of cortical areas in ADHD. Moreover, combined with previous findings that denser and stronger networks of cognitive functions were also observed in other psychiatric disorders (e.g., schizophrenia, major depression) when compared to healthy controls (Karyakina & Shmukler, 2021; Liang et al., 2018), future studies need to explore how the network of cognitive functions of ADHD relates to the networks of a community sample or specific psychiatric disorders other than ADHD.

Even though fewer and weaker connections were observed in the n-ADHD compared to the ADHD network, there are some consistent connections that were observed in both the ADHD and n-ADHD networks, including the connections between measures of selective attention and vigilance (nodes 1 – 5) and connections between measures of processing speed, flexibility, and fluency (nodes 7, 10, 14). The connections between selective attention and vigilance add evidence to the argument that different attention components are related in terms of behavioral performance as well as its neural basis (Angelelli et al., 2020; McDowd, 2007; Stuss & Alexander, 2007; Wilding, 2005). The correlations between processing speed, flexibility, and fluency add evidence to the notion that basic functions (e.g., processing speed) are substantially related to more complex cognitive functions (i.e., fluency and flexibility) and that training of processing speed may also improve performance on executive functions (Butzbach et al., 2019; Guo et al., 2021; Lin et al., 2016; Mohamed et al., 2021; Sheline et al., 2006; Takeuchi & Kawashima, 2012). In addition, the correlations between variables of the same test (e.g., TMT, WAFS, WAFV), and strong correlations between related functions (e.g., between selective attention and vigilance), may indicate some redundancy in lengthy test batteries and the possibility to tailor assessment batteries more efficiently to clinical and individual needs. We conclude that the possibility of shortening neuropsychological assessment batteries may be attractive to minimizing or avoiding fatigue (Feltmate et al., 2020; Luna et al., 2018; L. Tucha et al., 2017), increase compliance by examinees, and save valuable clinical resources in unnecessary administration, scoring, and interpretation of test data. Furthermore, shorter test batteries may have the advantages that existing norm data are more valid if applied to individual performance data that may not underly pronounced transfer effects in extensive test batteries.

Other than these consistent connections, most variables in the ADHD network were weakly or moderately correlated with each other, such as working memory, planning, response inhibition, and interference control. The position of these functions in the network is partly consistent with earlier findings that children with ADHD show deficits in several relatively independent neuropsychological functions, including working memory, inhibition, and response variability (Coghill et al., 2014), which have also been assessed in this study. However, some dependence between neuropsychological functions in weak to moderate size was shown earlier and is underlined by the fact that individuals with ADHD mostly show deficits in more than one of the functions assessed, for example, 46% of children with ADHD show impairments in at least two of six functions assessed by Coghill et al. (2014), and 81% of adults with ADHD show deficits in at least two of the 10 functions assessed by Guo et al. (2021). In a study on self-reported neuropsychological functioning, 80% of adults with ADHD reported deficits in at least two of eight aspects of functioning (Fuermaier et al., 2014). The differences in occurrence rates of neuropsychological impairments can be explained by various factors, as this may depend on the functions assessed in the respective test battery, the test characteristics, and the referral context. Moreover, the observed association between working memory and the variability of RT in the ADHD network extends the argument that slowed processing speed may be a cause for working memory deficits in ADHD (Karalunas & Huang-Pollock, 2013; Weigard & Huang-Pollock, 2017), by suggesting that it may be the variability of RT that causes impairments in working memory, not the slowed down responses. It may also serve as an explanation for why an earlier meta-analytic review revealed that slow RTs in ADHD may disappear after controlling for RT variability (Kofler et al., 2013). For clinical practice, we may conclude that task switching should be assessed separately in a comprehensive neuropsychological investigation because of the weak and few connections of this function with other functions that are commonly assessed.

Moreover, the highest expected influence of attention-related variables (e.g., RT of selective attention, RT of vigilance, and omissions of vigilance) in both the ADHD and the n-ADHD network stresses the central role of attention for a broad range of other neuropsychological functions. High expected influence of attention observed in this study provides new empirical evidence to the argument that basic cognitive functions are significantly associated with and contribute to the higher-order cognitive functions as suggested in numerous studies (Adams et al., 2011; Arciniegas et al., 2002; Butzbach et al., 2019; Felmingham et al., 2004; Guo et al., 2021; Mohamed et al., 2021). On the basis of the central role of attention in relation to other cognitive functions, clinicians may be advised to consider attention-related tests as the first choice when composing an assessment battery for the clinical evaluation of adult ADHD. Also, it could be speculated that improving attention abilities in the treatment of ADHD may secondarily also improve other cognitive functions that build upon attention. For example, methylphenidate (MPH) has been shown to be effective in improving attention abilities in patients with ADHD (Hadar et al., 2021; Pievsky & McGrath, 2018; Spencer et al., 2009; Tamminga et al., 2016; O. Tucha, Mecklinger, et al., 2006; O. Tucha, Prell, et al., 2006) and was also shown to be effective in improving the ability of higher-order cognitive functions, such as planning, memory, fluency, inhibition, and interference control (Abikoff et al., 2009; Fuermaier et al., 2017; Kobel et al., 2009; Rubio Morell & Hernández Expósito, 2019; Tamminga et al., 2016; Yang et al., 2012; Yildiz et al., 2011). Even though these studies do not provide evidence to the treatment mechanisms, the network structure of this study gives support to the notion that MPH may improve primarily attention functions which may positively affect a broad range of other cognitive functions secondarily. In this vein, other types of treatment for ADHD may show a similar mechanism in improving neuropsychological functions, such as cognitive training or biofeedback (Cortese et al., 2015; Monastra, 2005; Monastra et al., 2006; Sonuga-Barke et al., 2014; Stern et al., 2016).

Furthermore, to address the possibility that different weights for each test (e.g., more variables were extracted from tests for selective attention and vigilance compared to other tests) may bias the findings of centrality estimation, additional network analysis was performed based on averaged *Z*-scores per neuropsychological function. Results were comparable to our initial analysis, such as the strong connection between selective attention and vigilance, the strong connection between processing speed/flexibility and fluency, and the highest expected influence of selective attention and vigilance. These results support the reliability of our initial analysis based on multiple test scores per function (see Figures S3–S5 of the Supplemental Material). Finally, considering the nearly significant sex difference between the ADHD and n-ADHD group (*p* = .052), additional network analyses were carried out to examine the potential influence of sex (see Figures S6–S8 of the Supplemental Material). These additional analyses revealed no significant differences in global connectivity strength by sex, neither in the ADHD nor the n-ADHD group. Selective attention and vigilance still have the highest expected influence in the male and female networks within both the ADHD and *n*-ADHD groups. However, we noted that variables of selective attention (i.e., omissions and SDRT) have a seemingly higher expected influence in the male n-ADHD network compared to the female n-ADHD network, which needs replication on larger samples in future studies.

### Limitations and Future Directions

Several limitations of this study should be taken into account. First, effect sizes of network analyses cannot be calculated based on current statistical methodology. The magnitude of the findings, for example, to compare global connectivity or expected influence, would benefit the interpretation of the findings and their clinical implications. Second, it must be stressed that networks do not indicate causal relationships between functions. Even though “expected influence” may appear like directional paths, no such causal relationships can be inferred from networks (Bringmann et al., 2019; Dablander & Hinne, 2019). Third, the majority of individuals in the ADHD group (149 of 173) were diagnosed with the predominantly combined symptom presentation, leading to an unbalanced sample and potentially biased network estimation because of the potential different cognitive profiles across different subtypes of ADHD (LeRoy et al., 2019). Additional network analysis was performed on the individuals with the combined symptom presentation only (for details see Figures S9 and S10 of the Supplemental Material) and revealed no meaningful differences compared to the analysis of the entire group of patients with ADHD. Even though this study gives no indication for bias by ADHD subtype, future studies are needed on large samples of ADHD with sufficiently large numbers of the various ADHD symptom presentations to address this issue properly. Fourth, potential cognitive subtypes proposed in previous research (e.g., Roberts et al., 2017) may also affect the representativeness of our sample, which may require more thorough consideration on large samples in future studies. Fifth, future studies using network analysis should consider including a more comprehensive battery of neuropsychological functions that may be relevant in the assessment of ADHD. For example, timing, delay aversion, decision-making, and more memory functions (e.g., retrospective memory and prospective memory) have been included in many previous studies but were not included in this study because all assessments in this study were from a routine battery as part of the clinical protocol.

### Conclusions

This study is the first using network analysis to investigate the relationship between various neuropsychological functions in a large sample of clinically referred individuals at an ADHD outpatient clinic. Further strengths of this study are that it uses a naturalistic design, using data derived from the routine clinical practice of an ADHD clinic, as well as a clinical comparison group with similar characteristics in key clinical features which increases ecological validity. Network estimations and comparison revealed a denser and significantly stronger network of neuropsychological functions in the ADHD group compared to the n-ADHD group. The stronger and more interrelated network of neuropsychological functions observed in individuals with ADHD may be a starting point to identifying intertwined neuropsychological characteristics that are typical for ADHD. Furthermore, among the broad range of neuropsychological functions assessed, attention performance displayed the highest expected influence on other neuropsychological functions in both the ADHD and the n-ADHD network, which provides clinically relevant implications for the clinical assessment, treatment planning, and treatment evaluation of individuals with cognitive impairment.

## Supplemental Material

sj-docx-1-asm-10.1177_10731911221118673 – Supplemental material for Networks of Neuropsychological Functions in the Clinical Evaluation of Adult ADHD Click here for additional data file. Supplemental material, sj-docx-1-asm-10.1177_10731911221118673 for Networks of Neuropsychological Functions in the Clinical Evaluation of Adult ADHD by Nana Guo, Anselm B. M. Fuermaier, Janneke Koerts, Oliver Tucha, Norbert Scherbaum and Bernhard W. Müller in Assessment
