# Supplementary material for: Networks of Neuropsychological Functions in the Clinical Evaluation of Adult ADHD
Source: Assessment. 2022 Aug 29;30(6):1719–36. doi: 10.1177/10731911221118673 (PMC10363951; doi:10.1177/10731911221118673)
Supplement: sj-docx-1-asm-10.1177_10731911221118673 – Supplemental material for Networks of Neuropsychological Functions in the Clinical Evaluation of Adult ADHD [file sj-docx-1-asm-10.1177_10731911221118673.docx]

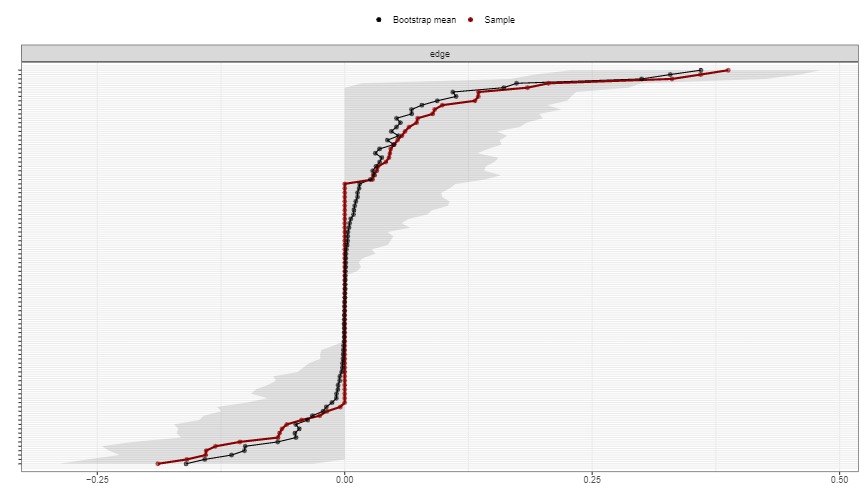


**Figure S1.** Bootstrapped CIs of estimated edge-weights for the estimated network of the ADHD group. *Note*. Each horizontal line represents one edge of the network, ordered from the edge with the highest edge-weight to the edge with the lowest edge-weight. The red line indicates the sample values of edge weights and the black line indicates the Bootstrap mean values of edge weights. The gray area indicates the bootstrapped CIs. The y-axis labels have been removed to avoid cluttering.


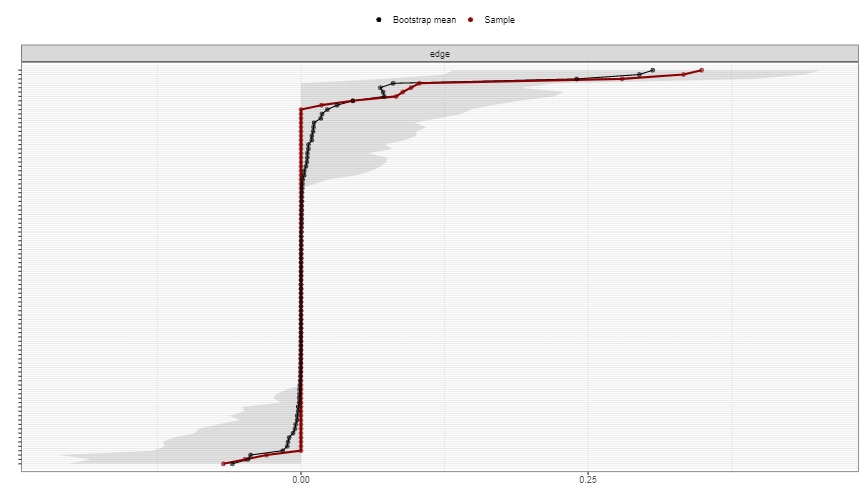


**Figure S2.** Bootstrapped CIs of estimated edge-weights for the estimated network of the n-ADHD group. *Note*. The gray area indicates the bootstrapped CIs. Each horizontal line represents one edge of the network, ordered from the edge with the highest edge-weight to the edge with the lowest edge-weight. The y-axis labels have been removed to avoid cluttering.


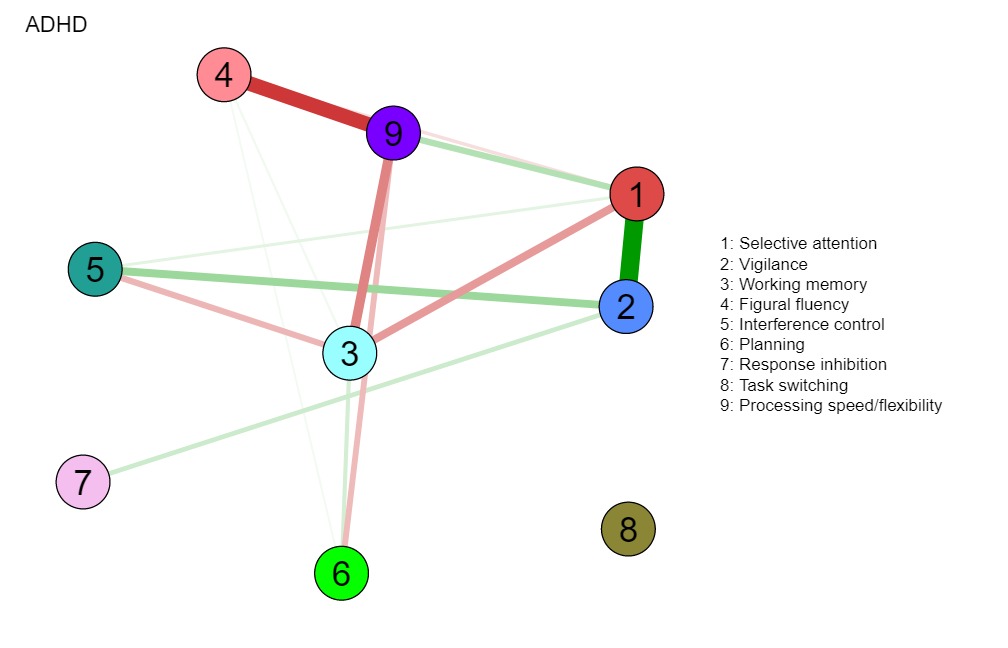


**Figure S3.** Network of neuropsychological functions for the ADHD group based on (averaged) Z-scores (N = 173).

*Note*. Nodes represent neuropsychological functions. Each neuropsychological function is presented in a different color. Edges connecting nodes represent the regularized partial Spearman correlations. Higher absolute correlations are represented with thicker and more saturated colored edges. Green edges indicate positive correlations, red edges indicate negative correlations.


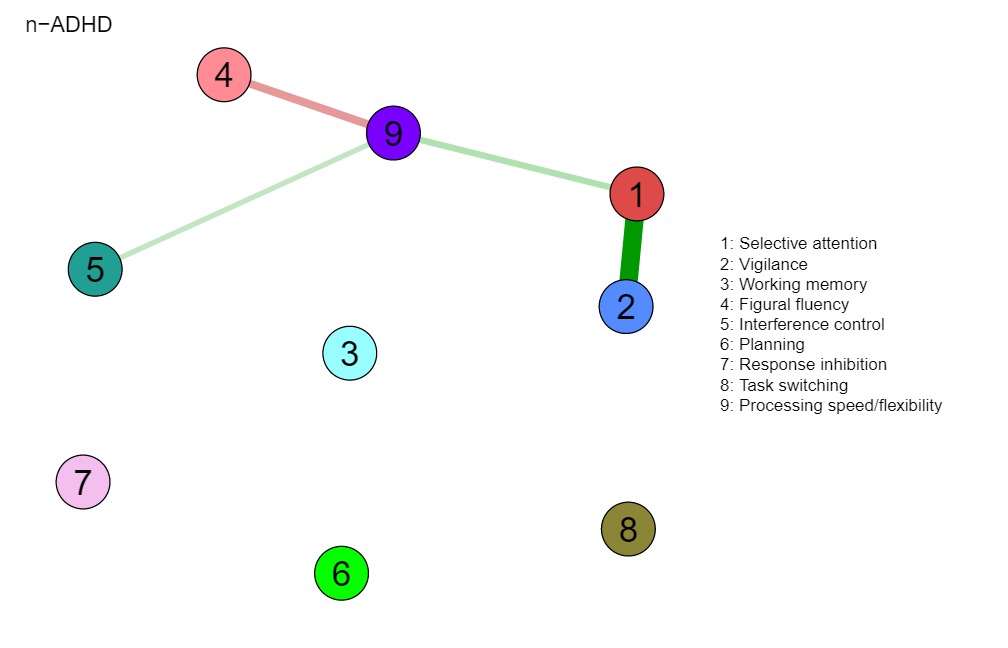


**Figure S4.** Network of neuropsychological functions for the n-ADHD group based on (averaged) Z-scores (N = 146).

*Note*. Nodes represent neuropsychological functions. Each neuropsychological function is presented in a different color. Edges connecting nodes represent the regularized partial Spearman correlations. Higher absolute correlations are represented with thicker and more saturated colored edges. Green edges indicate positive correlations, red edges indicate negative correlations.


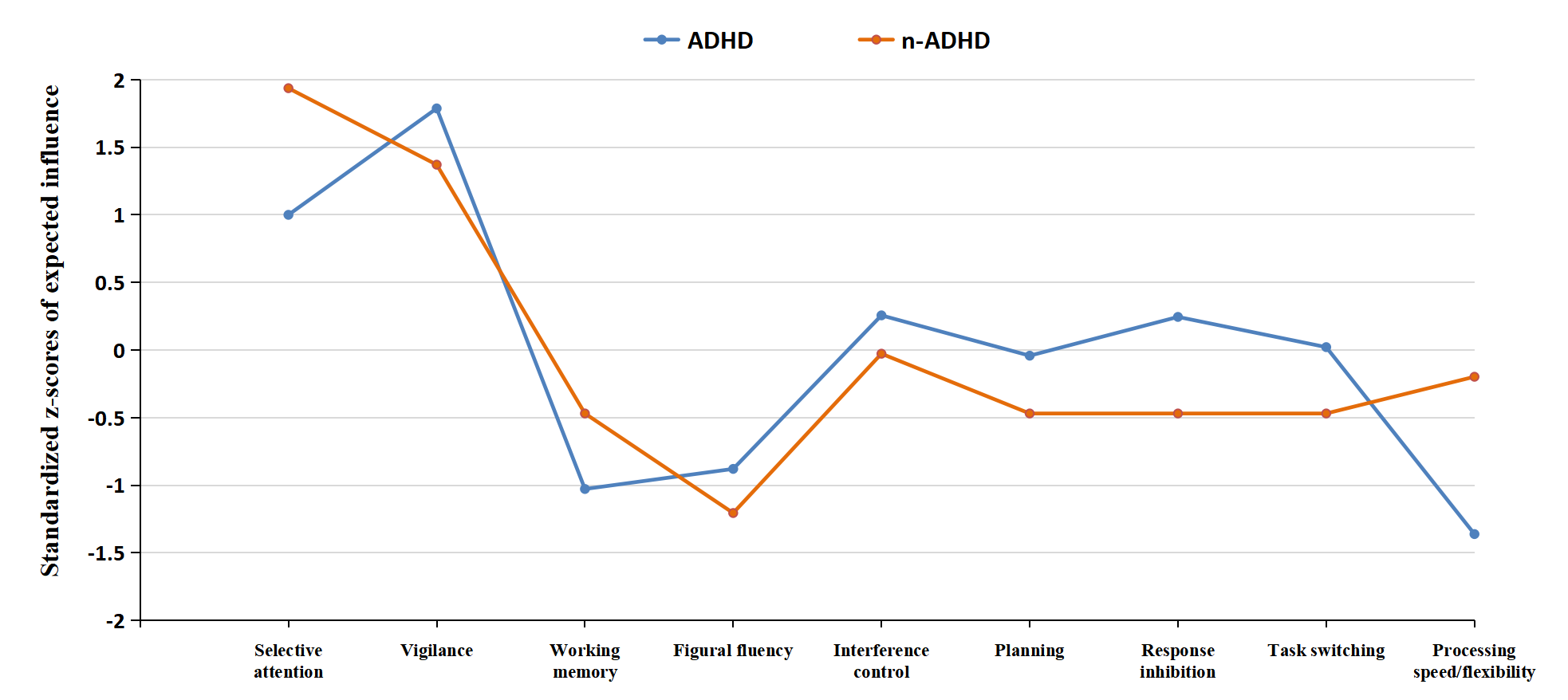


**Figure S5.** Node expected influence for the ADHD and n-ADHD networks based on (averaged) Z-scores.

*Note*. Higher standardized Z-scores indicate higher expected influence, and nodes with higher expected impact have closer and stronger relationships with other neuropsychological functions in the network.


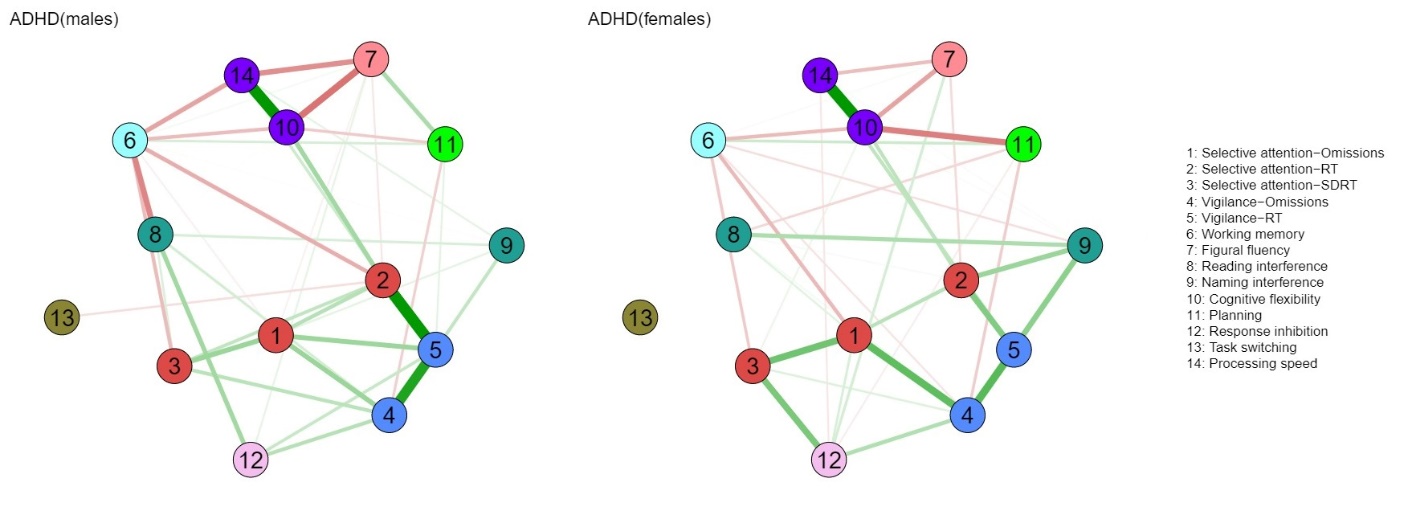


**Figure S6.** Networks of neuropsychological functions of individuals with ADHD, separately for males and females.

*Note*. Nodes represent neuropsychological test variables. Neuropsychological test variables stemming from the same test are presented in the same color. Edges connecting nodes represent the regularized partial Spearman correlations. Higher absolute correlations are represented with thicker and more saturated colored edges. Green edges indicate positive correlations, red edges indicate negative correlations.


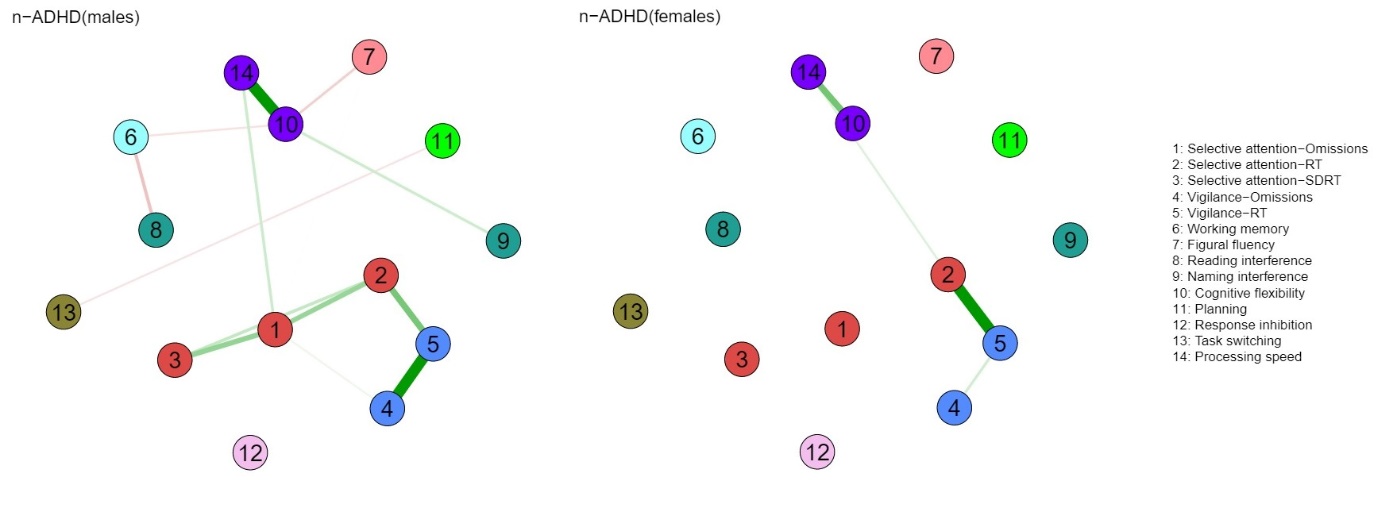


**Figure S7.** Networks of neuropsychological functions of individuals not diagnosed with ADHD, separately for males and females.

*Note*. Nodes represent neuropsychological test variables. Neuropsychological test variables stemming from the same test are presented in the same color. Edges connecting nodes represent the regularized partial Spearman correlations. Higher absolute correlations are represented with thicker and more saturated colored edges. Green edges indicate positive correlations, red edges indicate negative correlations.


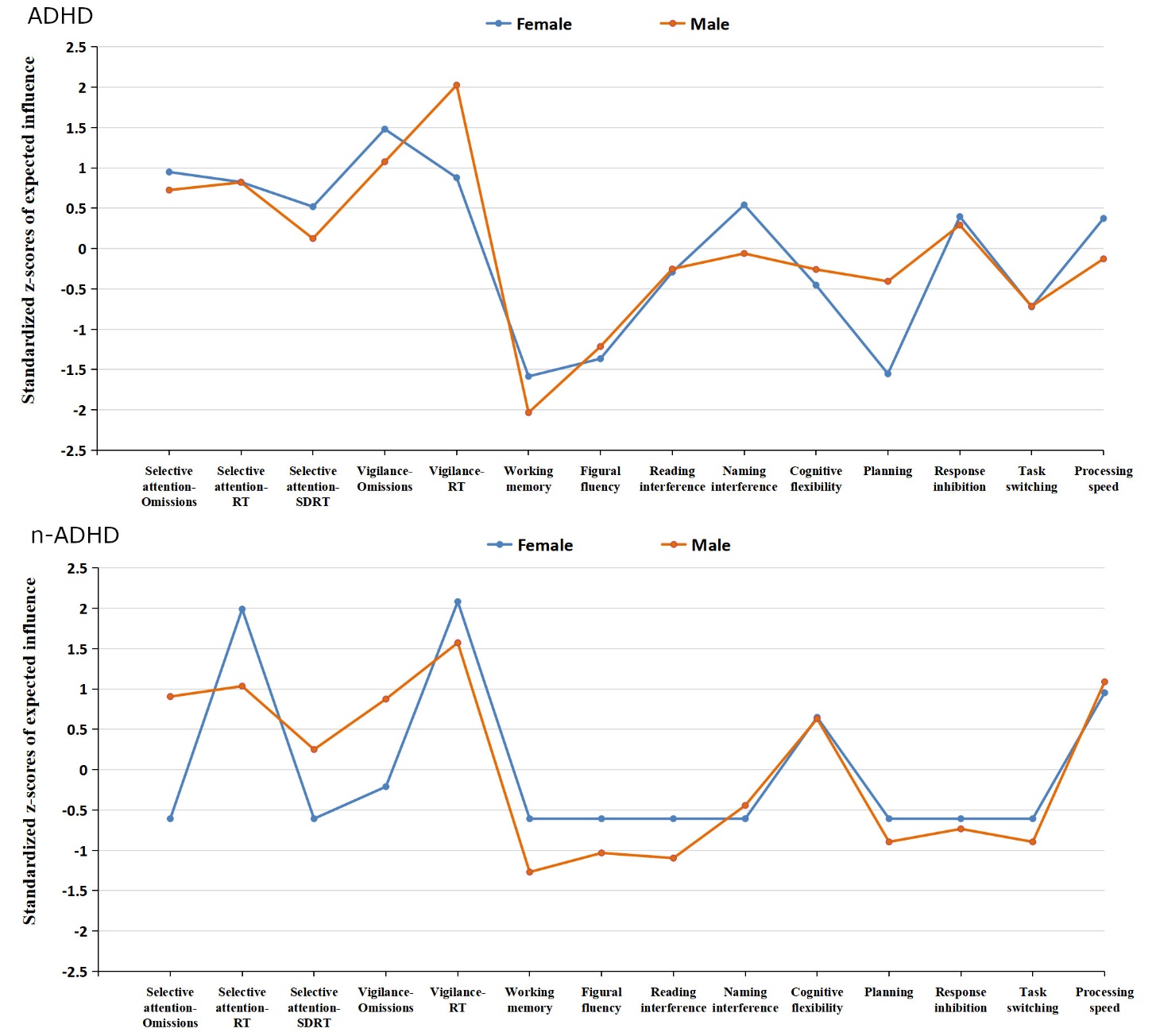


**Figure S8.** Node expected influence for the male and female networks in two groups.

*Note*. Higher standardized z-scores indicate higher expected influence, and nodes with higher expected impact have closer and stronger relationships with other neuropsychological test variables in the network.


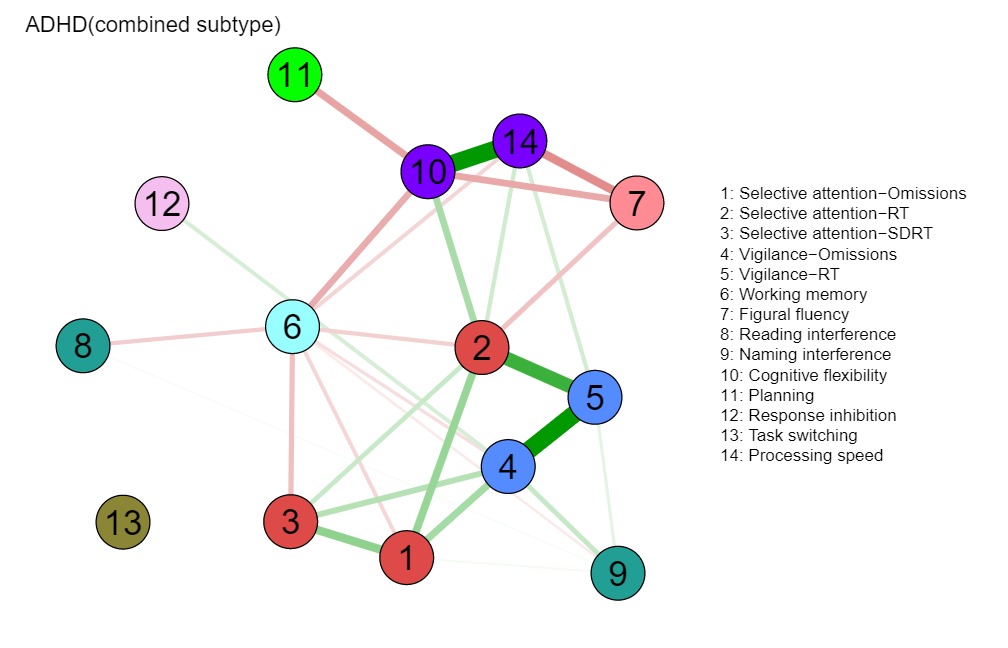


**Figure S9.** Network of neuropsychological functions for individuals with the combined symptom presentation of ADHD (N = 149).

*Note*. Nodes represent neuropsychological test variables. Neuropsychological test variables stemming from the same test are presented in the same color. Edges connecting nodes represent the regularized partial Spearman correlations. Higher absolute correlations are represented with thicker and more saturated colored edges. Green edges indicate positive correlations, red edges indicate negative correlations.


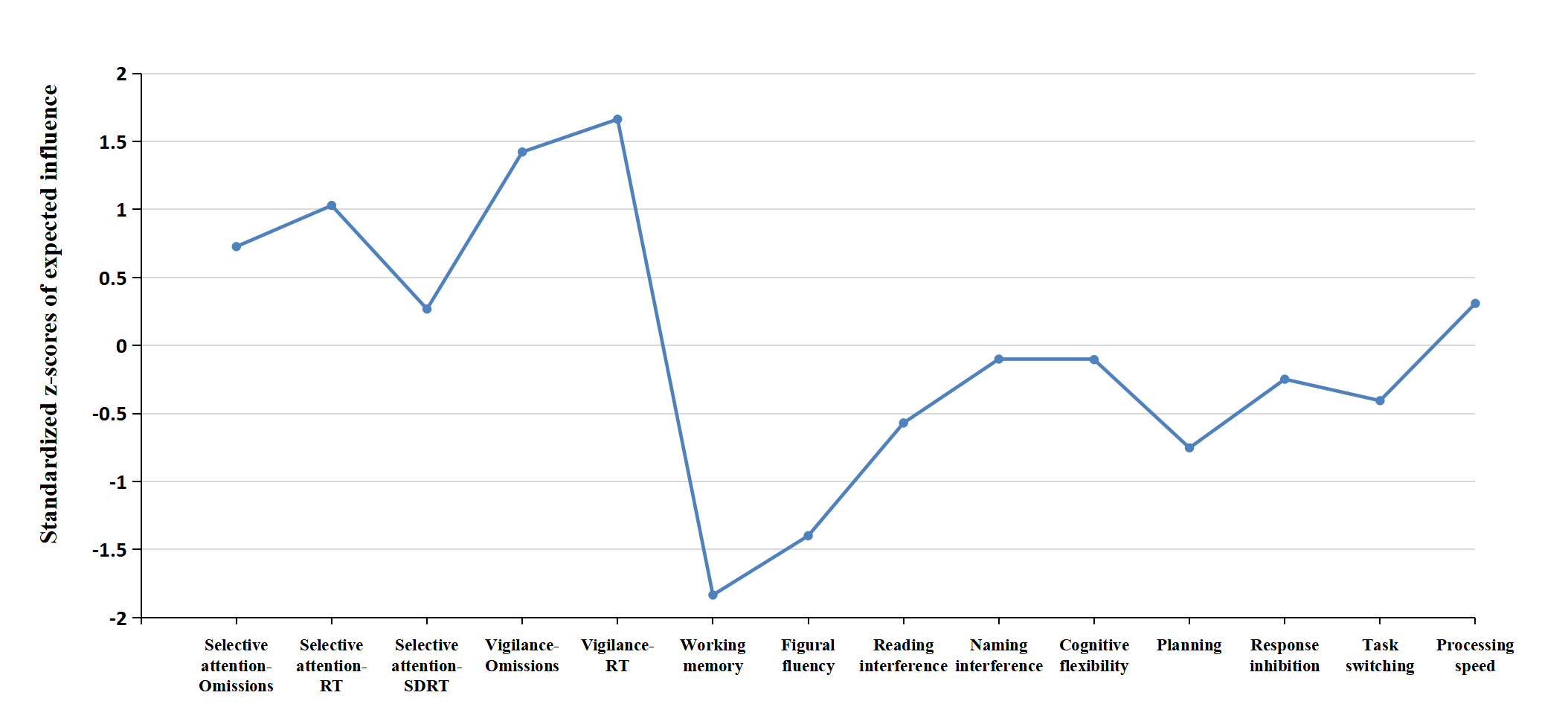


**Figure S10.** Node expected influence of the network of individuals with the combined symptom presentation of ADHD.

*Note*. Higher standardized Z-scores indicate higher expected influence, and nodes with higher expected impact have closer and stronger relationships with other neuropsychological test variables in the network.
